# Methotrexate-Associated Lymphoproliferative Disorder Presenting As Disseminated Metastatic Malignancy: A Case Report

**DOI:** 10.7759/cureus.115901

**Published:** 2026-09-07

**Authors:** Carolina Martins, Joana C Ramos, Pedro A Dias, Catarina Lucas

**Affiliations:** 1 Internal Medicine, Hospitais da Universidade de Coimbra, Coimbra, PRT; 2 Internal Medicine, Centro Hospitalar Universitário de Coimbra, Coimbra, PRT; 3 Internal Medicine, Centro Hospitalar e Universitário de Coimbra, Coimbra, PRT

**Keywords:** methotrexate, methotrexate-associated lymphoproliferative disorder, osteolytic lesions, spontaneous regression, systemic lupus erythematosus

## Abstract

Methotrexate-associated lymphoproliferative disorder (MTX-LPD) is a rare but relevant complication of long-term methotrexate (MTX) therapy that can closely mimic infection or disseminated malignancy.

We report the case of a 65-year-old woman with a 10-year history of systemic lupus erythematosus (SLE) treated with MTX, who presented with progressive back pain, constitutional symptoms, pancytopenia, and extensive multiorgan disease. Fluorodeoxyglucose positron emission tomography (FDG PET) demonstrated widespread hypermetabolic lesions involving the lungs, liver, left adrenal gland, and skeleton, raising strong suspicion of disseminated metastatic malignancy. Two ultrasound-guided liver nodule biopsies were inconclusive, with no evidence of malignancy. Given the absence of histopathological confirmation and her prolonged MTX exposure, MTX-LPD was suspected, and MTX was discontinued. No oncologic treatment was initiated. Four months later, the patient had achieved complete clinical resolution of her symptoms, and follow-up imaging showed near-complete regression of the pulmonary, hepatic, adrenal, and most skeletal lesions.

## Introduction

Methotrexate (MTX) is among the most widely prescribed disease-modifying antirheumatic drugs and remains the cornerstone in the treatment of rheumatoid arthritis and other autoimmune diseases owing to its well-established efficacy, favorable safety profile, and affordability [1]. Although generally well tolerated, long-term MTX therapy may be associated with a range of adverse effects, including hepatotoxicity, myelosuppression, opportunistic infections and, more rarely, methotrexate-associated lymphoproliferative disorder (MTX-LPD) [2-4].

MTX-LPD is classified as an other iatrogenic immunodeficiency-associated lymphoproliferative disorder (OIIA-LPD) [2,3]. Its clinical presentation is highly heterogeneous and may mimic infection, autoimmune disease activity, or metastatic malignancy, often posing a significant diagnostic challenge [4-6]. Recent reports describing atypical presentations, including diffuse pulmonary ground-glass opacities and isolated vertebral involvement, further illustrate the broad clinical spectrum of this condition [7,8].

Unlike most lymphomas, MTX-LPD may undergo spontaneous regression following MTX withdrawal alone, making early recognition crucial [2,5,8]. We report the case of a patient with systemic lupus erythematosus (SLE) who developed extensive multiorgan disease while receiving long-term MTX, with MTX-LPD considered the most likely diagnosis. The initial clinical and imaging findings suggested disseminated metastatic cancer. This case highlights the importance of considering MTX-LPD in patients receiving long-term MTX therapy with unexplained systemic disease [2,5,8]. 

## Case presentation

A 65-year-old woman with a 10-year history of SLE, treated with MTX (20 mg/week), hydroxychloroquine (200 mg/daily), and prednisolone (5 mg/daily), presented with progressively worsening back pain over a two-week period. Prior to symptom onset, she was fully independent in all activities of daily living. Owing to persistent back pain, a bone scintigraphy was performed, revealing multiple osteolytic lesions involving several thoracic vertebrae, the right clavicle and two ribs bilaterally. She was subsequently referred for further evaluation.

Over the preceding month, she had also developed progressive fatigue, exertional dyspnea, anorexia, and unintentional weight loss. She denied fever, night sweats, cough, or other constitutional symptoms. Laboratory investigations revealed new-onset pancytopenia, with a hemoglobin level of 5.8 g/dL, a leukocyte count of 1.6 × 10⁹/L, and a platelet count of 66 × 10⁹/L. Liver enzymes and C-reactive protein (7.61 mg/dL) were also elevated (Table 1), prompting hospital admission for further investigation.

**Table 1:** Results of the serum/blood tests done at the time of presentation and at four months after MTX withdrawal WBC: White blood cells, LDH: Lactate dehydrogenase; ALT: Alanine aminotransferase; AST: Aspartate aminotransferase; ALP: Alkaline phosphatase; GGT: Gamma-glutamyl transferase; ESR: Erythrocyte sedimentation rate; HIV: Human immunodeficiency virus; CMV: Cytomegalovirus; EBV: Epstein-Barr virus; MTX: Methotrexate

Laboratory parameters	At the beginning of the symptoms	4 months after MTX withdrawal	Reference range
Hemoglobin (g/dL)	5.8	10.3	12.0-15.6
Hematocrit (%)	17.6	31.0	35-45.5
Mean corpuscular volume (fL)	80.6	85.4	80-99
WBC (x10^9^/L)	1.6	6.4	3.60-10.5
Neutrophils (x10^9^/L)	1.44	5.35	1.50-7.70
Lymphocytes (x10^9^/L)	0.03	0.49	1.10-4.40
Platelets (x10^9^/L)	66	247	140-385
LDH (U/L)	651	270	<247
ALT (U/L)	66	23	<34
AST (U/L)	54	21	<31
ALP (U/L)	165	74	30-120
GGT (U/L)	82	106	<38
Total bilirubin (mg/dL)	0.3	0.5	0.2-1.2
C-reactive protein (mg/dL)	7.61	0.55	< 0.50
ESR (mm/h)	76	65	1-20
Anti-dsDNA antibodies (IU/mL)	3.88	-	<7.0
C3 complement (g/L)	0.96	-	0.83-1.93
C4 complement (g/L)	0.16	-	0.15-0.57
Ferritin (ng/mL)	515.8	-	30-300
Vitamin B12 (pg/mL)	677	-	187-883
Folic acid (ng/mL)	8.2	-	>3.5
D-dimers (ng/mL)	402	-	<500
Fibrinogen (mg/dL)	322	-	200-500
Haptoglobin (g/L)	1.6	-	0.30-2.0
Triglycerides (mg/dL)	149	-	43.8-195.1
Serum protein electrophoresis	Normal	-	-
Peripheral blood immunophenotyping	Normal	-	-
HIV serology	Negative	-	-
Hepatitis B serology	Negative	-	-
Hepatitis C serology	Negative	-	-
CMV serology	Negative	-	-
EBV serology	Suggestive of previous infection	-	-
Parvovirus serology	Negative	-	-
Interferon Gamma Release Assay	Negative	-	

An extensive diagnostic workup excluded active SLE, hemolysis, nutritional deficiencies, and hemophagocytic lymphohistiocytosis (HLH) and macrophage activation syndrome (MAS). Infectious screening, including human immunodeficiency virus (HIV), hepatitis B and C, cytomegalovirus (CMV), parvovirus, and tuberculosis, was negative, while Epstein-Barr virus (EBV) serology indicated previous infection. Serum protein electrophoresis, immunofixation, and peripheral blood immunophenotyping were unremarkable, with no evidence of monoclonal gammopathy or overt hematological malignancy (Table 1). Blood and urine cultures were also negative.

Contrast-enhanced whole-body CT demonstrated multiple bilateral pulmonary nodules, hepatomegaly with numerous hepatic nodules, and a new osteolytic lesion (Figure 1). These findings raised strong suspicion of disseminated metastatic malignancy from an unknown primary tumor. Subsequent fluorodeoxyglucose positron emission tomography (FDG PET) revealed extensive hypermetabolic pulmonary lesions, lymphatic involvement predominantly involving the left lung, with extensive reticular opacities and interlobular septal thickening, as well as hepatic, left adrenal, and diffuse skeletal involvement, as shown in Figure 2.

**Figure 1:**
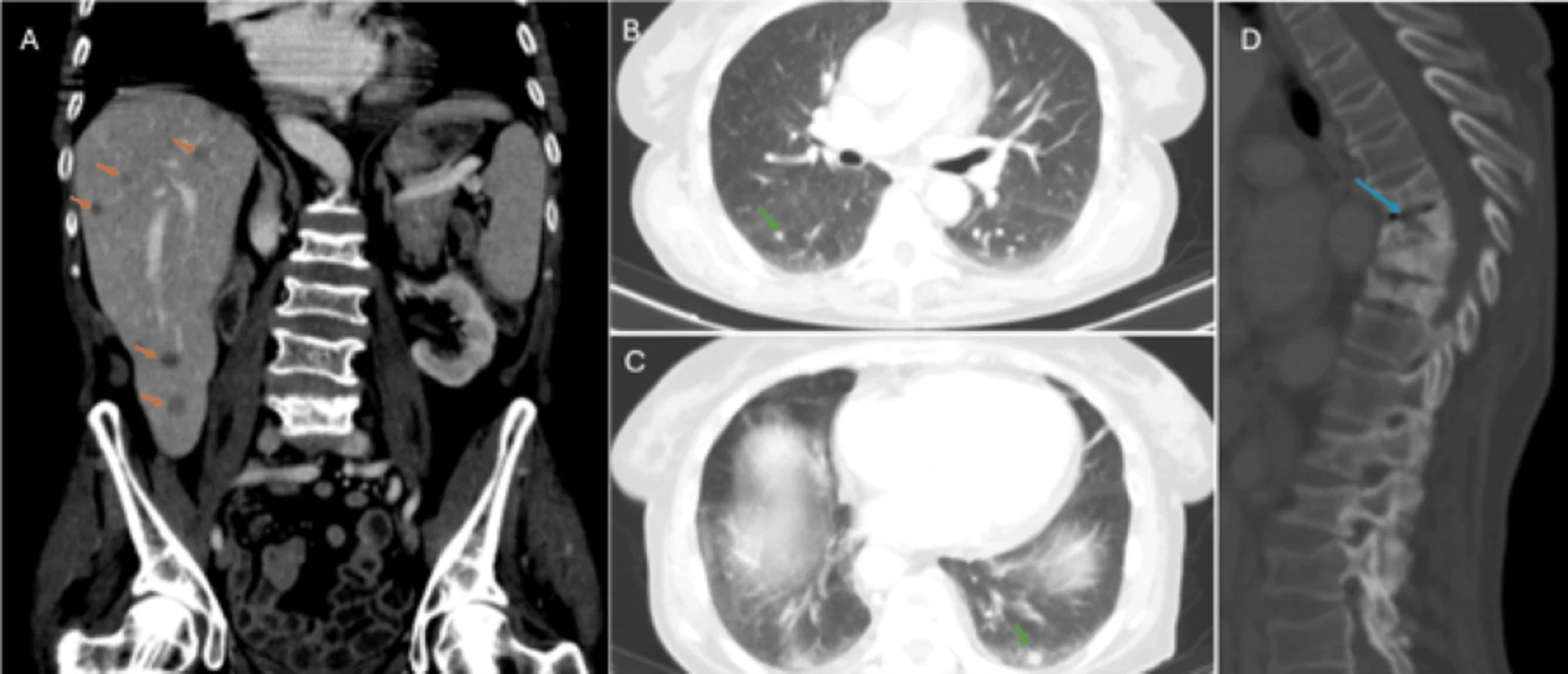
CT findings at presentation (A) Hepatomegaly with multiple hepatic nodules (orange arrows); (B, C)Figures B and C show two pulmonary nodules (green arrows); (D) New osteolytic lesion in D7 (blue arrow)

**Figure 2:**
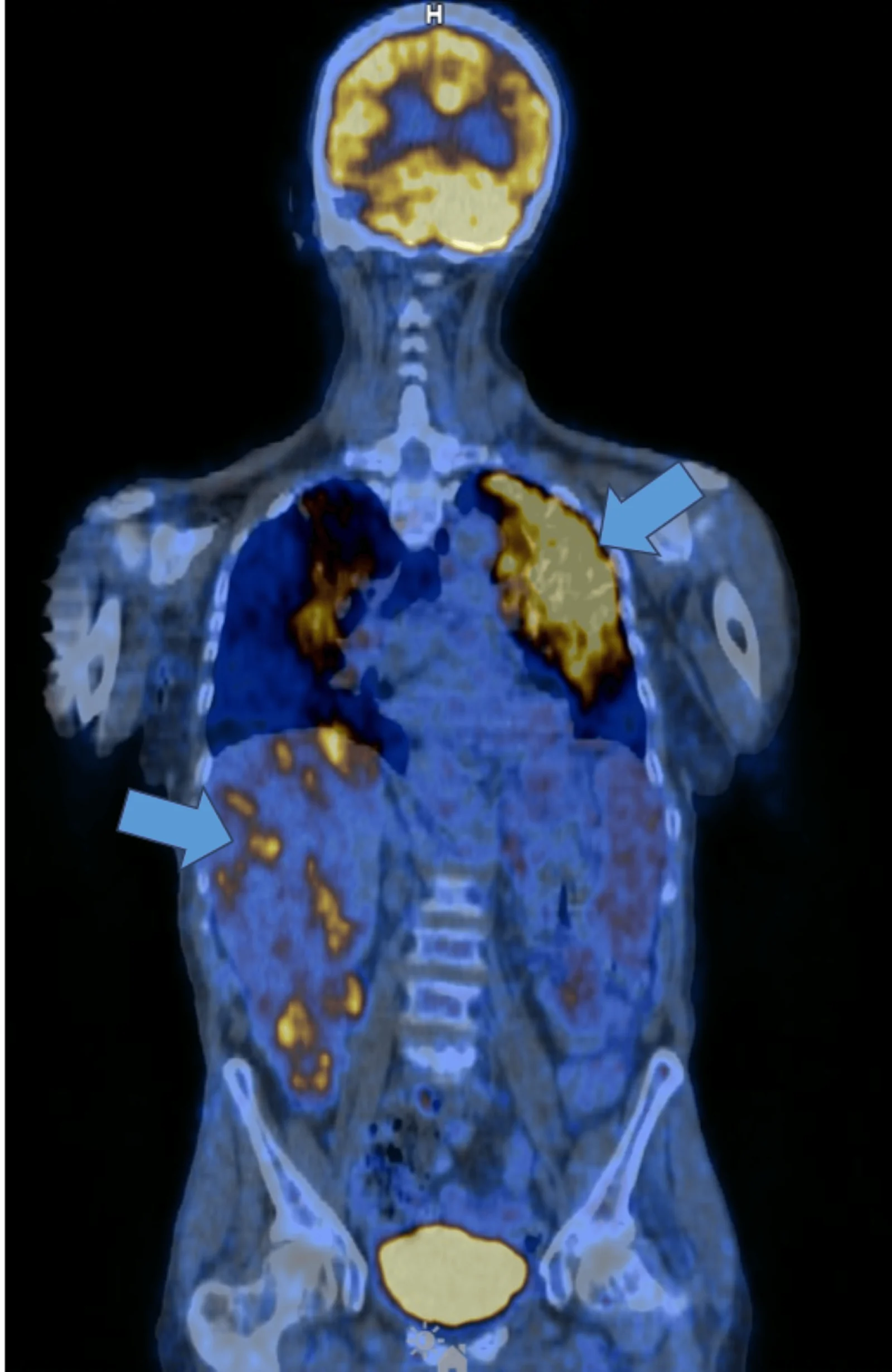
FDG PET findings before MTX withdrawal Arrows indicate hypermetabolic foci of increased 18F-FDG uptake, suggestive of neoplastic involvement. FDG PET: Fluorodeoxyglucose positron emission tomography; MTX: Methotrexate; FDG: Fluorodeoxyglucose

Ultrasound-guided biopsy of two distinct hepatic lesions was performed on two separate occasions. The first histological examination revealed a scarce inflammatory infiltrate with scattered CD68-positive macrophages, with no signs of malignancy. The second biopsy revealed preserved hepatic architecture with no evidence of neoplastic involvement. In the absence of histopathological confirmation of malignancy/LPD despite extensive radiological disease, and given the patient’s long-term MTX exposure, MTX-LPD was considered, and it was discontinued.

The patient was managed conservatively with close outpatient follow-up and received no oncologic treatment, as she declined further invasive procedures, given her progressive clinical improvement over the following months. Four months after MTX withdrawal, she reported complete resolution of her symptoms and had achieved marked clinical improvement. Follow-up FDG PET demonstrated near-complete resolution of the pulmonary, hepatic, adrenal, and skeletal lesions (Figure 3). MTX-LPD was considered the most likely diagnosis based on the clinical context, exclusion of malignancy in two biopsies, unremarkable blood work and subsequent regression after MTX withdrawal.

**Figure 3:**
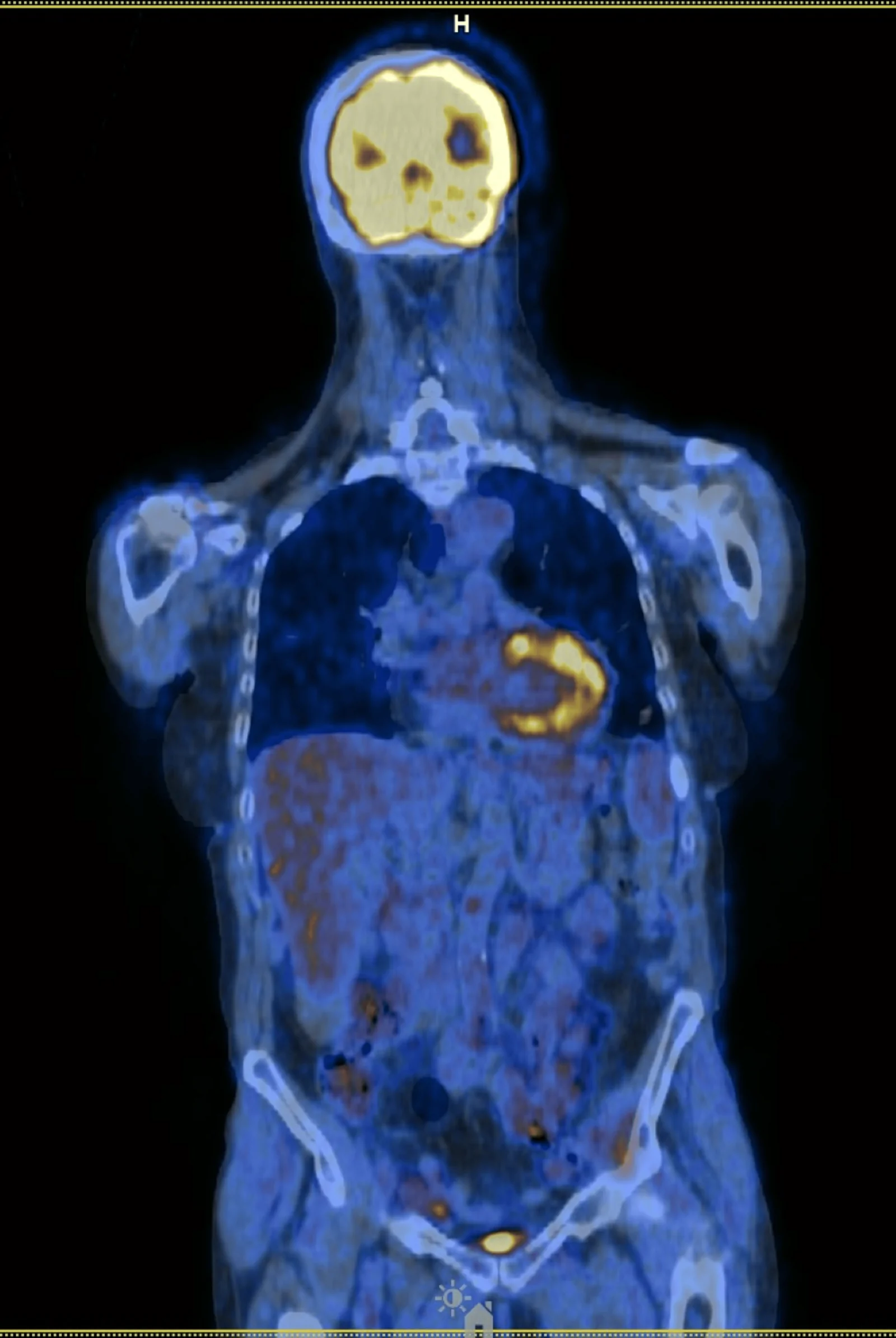
FDG PET findings after MTX withdrawal Previous areas of increased tracer uptake have resolved, with only physiological tracer uptake remaining. FDG PET: Fluorodeoxyglucose positron emission tomography; MTX: Methotrexate

## Discussion

MTX-LPD is an uncommon but clinically important complication of long-term MTX therapy. Although it is most frequently reported in patients with rheumatoid arthritis, it has also been described in association with other autoimmune diseases, including SLE [1-6].

This case highlights the diagnostic challenge posed by MTX-LPD, particularly when it presents with widespread multisystem involvement. The combination of nonspecific constitutional symptoms, pancytopenia, and extensive radiological abnormalities initially raised strong suspicion of disseminated metastatic malignancy. Recognition of the patient’s prolonged MTX exposure as a potential underlying cause was pivotal to establishing the correct diagnosis and guiding appropriate management [2,5]. 

A particularly unusual feature of this case was the extensive bone involvement. Although extranodal disease is common in MTX-LPD, skeletal involvement is relatively uncommon, and reports describing multiple osteolytic lesions are particularly rare. Hirata et al. reported a case presenting with an osteolytic vertebral lesion, illustrating how MTX-LPD can closely mimic metastatic bone disease [7]. Our patient demonstrated far more extensive skeletal involvement, with multiple osteolytic lesions accompanied by pulmonary, hepatic, and adrenal lesions, further expanding the recognized spectrum of this condition. Similarly, recent reports have described atypical pulmonary manifestations, further highlighting the diverse clinical and radiological presentations of MTX-LPD [8].

One of the defining characteristics of MTX-LPD is its potential for spontaneous regression following MTX withdrawal alone. Consistent with this, our patient experienced complete resolution of constitutional symptoms, accompanied by marked regression of the pulmonary, hepatic, and adrenal lesions after MTX discontinuation, without requiring chemotherapy. Interestingly, the bone lesions exhibited a heterogeneous pattern of response. While the lesions involving the ribs, clavicle, pelvis and femur regressed completely, the vertebral lesions persisted on follow-up FDG PET and continued to demonstrate increased 18F-FDG uptake, despite remaining morphologically stable. 

Although histopathological confirmation was not achieved, the marked clinical and radiological regression following MTX withdrawal, together with the exclusion of alternative diagnoses, strongly supported the diagnosis of MTX-LPD. 

## Conclusions

This case highlights the importance of considering MTX-LPD in patients receiving long-term MTX who present with unexplained systemic symptoms and extensive multisystem disease mimicking disseminated malignancy. Despite the absence of histopathological confirmation, the exclusion of alternative diagnoses and the marked clinical and radiological regression following MTX withdrawal strongly supported MTX-LPD as the most likely diagnosis.

This case also emphasizes the potential for significant disease regression following MTX withdrawal alone. Awareness of this potentially reversible complication is essential to ensure timely recognition, appropriate management, and adequate follow-up.
